# MCP extensors respond faster than flexors in individuals with severe-to-moderate stroke-caused impairment: Evidence of uncoupled neural pathways

**DOI:** 10.3389/fneur.2023.1119761

**Published:** 2023-03-22

**Authors:** Dongwon Kim, Raziyeh Baghi, Kyung Koh, Li-Qun Zhang

**Affiliations:** ^1^Department of Physical Therapy and Rehabilitation Science, University of Maryland, Baltimore, MD, United States; ^2^Department of Bioengineering, School of Engineering, University of Maryland, College Park, MD, United States; ^3^Department of Orthopedics, University of Maryland, Baltimore, MD, United States

**Keywords:** MCP joints, flexion synergy, hand dexterity, extensor, stroke

## Abstract

Damage in the corticospinal system following stroke produces imbalance between flexors and extensors in the upper extremity, eventually leading to flexion-favored postures. The substitution of alternative tracts for the damaged corticospinal tract is known to excessively activate flexors of the fingers while the fingers are voluntarily being extended. Here, we questioned whether the cortical source or/and neural pathways of the flexors and extensors of the fingers are coupled and what factor of impairment influences finger movement. In this study, a total of seven male participants with severe-to-moderate impairment by a hemiplegic stroke conducted flexion and extension at the metacarpophalangeal (MCP) joints in response to auditory tones. We measured activation and de-activation delays of the flexor and extensor of the MCP joints on the paretic side, and force generation. All participants generated greater torque in the direction of flexion (*p* = 0.017). Regarding co-contraction, coupled activation of the extensor is also made during flexion in the similar way to coupled activation of the flexor made during extension. As opposite to our expectation, we observed that during extension, the extensor showed marginally significantly faster activation (*p* = 0.66) while it showed faster de-activation (*p* = 0.038), in comparison to activation and de-activation of the flexor during flexion. But movement smoothness was not affected by those factors. Our results imply that the cortical source and neural pathway for the extensors of the MCP joints are not coupled with those for the flexors of the MCP joints.

## Introduction

It is common that stroke survivors have more strength and better volitional control of flexors, in comparison to extensors, at distal joints including the finger joints. In our daily life, we observe that distal joints in the upper extremity of stroke survivors with severe-to-moderate impairment tend to be in the flexion posture, rather than in the neutral or extension posture. Hand opening is more difficult for stroke survivors than hand closing (1–3).

Though mechanical constraints including muscle shortening and spasticity contribute to the asymmetry between flexors and extensors, neurological constraints are also a substantial contributor (4). One possible neurological reason of flexion-favored postures is the flexion synergy that links shoulder abductor and flexor with the flexors of the elbow, wrist and fingers (3). More distal joints including the wrist and finger joints are typically under the influence of the movement or posture of more proximal joints following stroke (5). Stroke binds muscles across the upper extremity into a small number of groups and the muscles in a group tend to concurrently activate (6–8). Normal hand manipulation requires lifting the arm and simply lifting the arm overcoming the gravitational force causes activation of the shoulder abductor and flexor, directly leading to flexing motion of distal joints. Indeed several researchers reported that arm support contributes to a decrease in the flexion synergy (1, 9, 10).

Another possible neurological reason of flexion-favored postures is increased activation of flexors during voluntary extension (4, 11). Extension torque produced is canceled out by flexion torque through excessive activation of flexors during voluntary extension. Indeed, co-activation of the flexors and extensors of the metacarpophalangeal (MCP) joints during voluntary MCP joint extension appears (4). A study reported that anesthesia of the flexors is effective in decreasing flexion torque produced during voluntary MCP extension, implying excessive flexor activation (4).

The common source of the flexion synergy and excessive activation of flexors during voluntary extension in the upper extremity might be the loss of corticospinal system input. The occurrence of a stroke can damage the corticospinal tract (CST) (12–16). Alternative pathways to the damaged CST include the ipsilateral corticospinal tract (17), vestibulospinal tract (18) and reticulospinal tract (RST) (12). In particular, use of the RST facilitates flexors while suppressing extensors (19, 20). This pathway excites the resting potential of the motoneurons closer to their thresholds *via* interneuronal excitation (21). Indeed RST upregulation occurs in the chronic phase (22). Then, a question arises here: are flexors and extensors activated and de-activated in a synchronized manner in the presence of the loss of corticospinal system input? Or are flexors and extensors controlled separately while extensor weakness is the main reason of asymmetry between flexors and extensors (4, 23).

In this study, we had participants with hemiplegic stroke respond to auditory tones by flexing and extending the MCP joints. The hand, in particular the MCP joints, can be regarded as a crucial component for upper-extremity dexterity. We measured response times of the MCP joints on the paretic side as well as their force generation and corresponding muscle activity. A previous study investigated response time of the paretic wrist joint and corresponding muscle activity during flexion and extension (24). But wrist and finger movements are made through different cortical activation (25), leading to a different recovery time (26). It would be worth revisiting neural antagonism between flexors and extensors by investigating MCP joint movement. Despite the need of a neurophysiological or imaging demonstration, Fugl-Meyer scores provide provides an indirect probe of the nervous system (26, 27). Fugl-Meyer scores could be indicative of the extent of CST integrity. We strived to investigate how differently flexors and extensors react depending on Fugl-Meyer scores.

## Methods

### Participants

A total of seven hemiplegic male volunteers post stroke [age: 61.57 ± 12.03 (SD) years; impaired side (L/R): 4/3, time since stroke: 7.63 ± 6.51 (SD) month] participated in the study. The inclusion criteria were moderate-to-severe upper extremity impairment [upper extremity Fugl-Meyer (UEFM) score < 43/66 (28)] and sufficient cognitive/language abilities to follow instructions during the experiment (Mini-Mental Status Score >22). We excluded volunteers who had severe shoulder pain, relevant musculoskeletal injury, or fixed contraction deformity in the upper extremity. None of the participants received pharmacological medications for spasticity and tone (i.e., Botulinum toxin injection to the upper limb) in the 5 months before the experiment. Each participant gave written informed consent approved by the Institutional Review Board of the University of Maryland, Baltimore. Table 1 presents the demographics of participants.

**Table 1:** Demographics of participants.

**Subject**	**Sex**	**Age**	**Paretic side**	**UEFM score**
*S1*	Male	53	Left	34
*S2*	Male	54	Right	29
*S3*	Male	61	Left	36
*S4*	Male	40	Left	10
*S5*	Male	77	Left	10
*S6*	Male	47	Right	26
*S7*	Male	72	Right	26

UEFM stands for upper-extremity Fugl-Meyer.

### Procedure

Participants sat on a height-adjustable chair with a back support and placed their paretic hand on a rotatable rigid plate that was connected to an electrical motor with an encoder (Maxon Brushless EC60, Sachseln, Switzerland). A torque transducer (Transducer Techniques TRT-100, Temecula, USA) was placed between the plate and motor. The MCP joints of the digits II-V were aligned with the rotation axis of the plate. The hand and forearm were supported being tightly fixed to the device using rigid mechanical blocks with cushion and Velcro straps. The arm posture was maintained with shoulder adduction of 45°, shoulder flexion of 45°, elbow flexion of 90°, and wrist flexion of 0°, respectively. Wireless EMG electrodes (Delsys, Boston, USA) were placed on the flexor digitorum superficialis (FDS) and extensor digitorum superficialis (EDS) muscles.

The experiment consisted of two sessions. The first session was to evaluate the ability of participants to flex and extend the MCP joints voluntarily. Participants were instructed to move their MCP joints back and forth as much as possible. The hand plate connected with the motor was backdriveable while it measured the angle of the MCP joints of the digits II–V.

The second session was to determine timing and muscle activity during MCP joint flexion and extension. The MCP joints of the digits II-V were passively locked by the motorized resistance when the joints were in the neutral position. Participants were first requested to flex and relax the MCP joints against motorized resistance in response to auditory tones. Three pairs of tones were given. Participants were asked to flex maximally, as quickly as possible, in response to the first tone of each pair, and relax as quickly as possible after the second tone. These pairs were placed 20 s apart to alleviate fatigue, and the first and second tones of the three pairs were gapped by 3, 2, and 4 s, respectively, to reduce the learning effect on timing. Verbal cues were presented prior to those three trials to get the participant ready to respond. Next participants were requested to extend and relax the MCP joints against motorized resistance in response to auditory tones. Three pairs of tones were given as well. Each participant was given to an enough practice period to get familiar to the instruction and reduce the effect of learning in responding to the auditory cues.

Data acquisition of joint angle and EMG signals were conducted in a LabVIEW environment. The sampling rate was set at 1,000 Hz.

### Analysis

**Figure 1:**
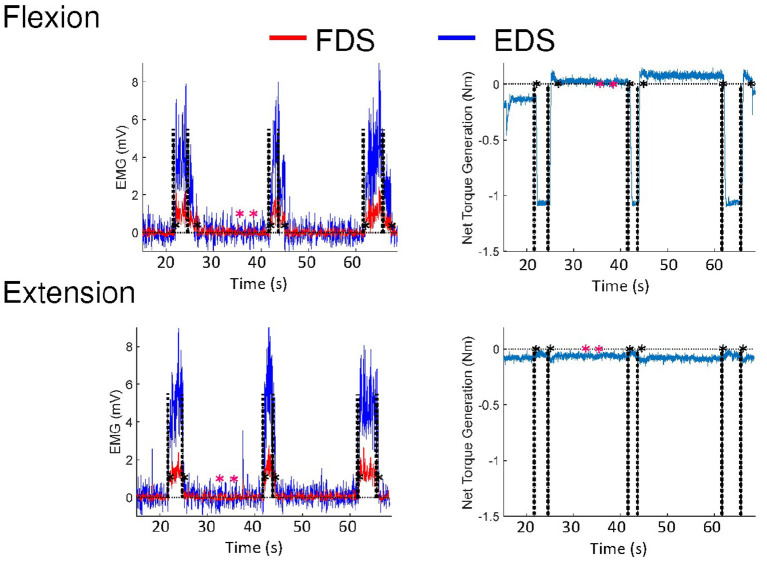
EMG and torque responses of a representative subject during voluntary flexion and extension, respectively. Black dashed lines indicate the times points of auditory tones occurring. The black asterisks indicate the time points that are registered by responses of the subjects versus a predefined threshold. The predefined threshold (3 standard deviations above the mean of EMG during the rest period) is based on the rest period defined by the pink asterisks. The subject involuntarily contracted the MCP flexor so the torque at the beginning was not 0.

The delays were determined by the time gap between the occurrences of the auditory tones and initiation/termination of muscle activation. Delays in initiation and termination of FDS and EDS were evaluated using EMG responses versus a predefined threshold (3 standard deviations above the mean of EMG during the rest period) (29), as seen in Figure 1. EMG signals were low-pass filtered at 225 Hz, rectified and low-pass filtered at 10 Hz using MATLAB (MathWorks, Natick, MA) to produce linear envelopes (LEs) (29). EMG LEs were subtracted by the mean of the EMG LE during the rest period.

To quantify the degree of activation of the antagonist muscle while the agonist muscle is voluntarily activated, antagonist activation ratios were calculated, respectively, as a ratio of the degree of activation of FDS or EDS when they are the antagonist muscle to that of FDS or EDS when they are the agonist muscle. The mean of the EMG LE of the agonist muscle (EDS or FDS) between the initiation and termination of the agonist muscle for each trial was obtained, while the mean of the EMG LE of the antagonist muscle was obtained during the same time period at each trial, during flexion and extension, respectively. The antagonist activation ratios were computed for each trial by dividing the mean of the EMG LE of the antagonist muscle for a trial by the mean of the EMG LE of the antagonist muscle when it was the agonist muscle for the corresponding trial.

Torque generation was also considered by averaging raw torque values from the torque transducer between the time points when the response of the agonist muscle (EDS or FDS) goes beyond and down the predefined threshold, during voluntary flexion or extension for each trial, respectively.

The spectral arc length was employed to evaluate the smoothness of the movement of the MCP joints (30, 31). This dimensionless metric is able to reflect movement intermittency, while minimizing the influence of movement amplitude or duration. Computation of the spectral arc length was carried out based on the angular velocity profile of the MCP joints ω_*MCP*_(*t*) as


(1) $$n_{sal}≜-\int_{0}^{\omega_{c}}\sqrt{{\left(\frac{1}{\omega_{c}}\right)}^{2}+{\left(\frac{d{\hat{W}}_{MCP}\left(\omega \right)}{d\omega }\right)}^{2}}d\omega ,$$

(2) $${\hat{W}}_{MCP}\left(\omega \right)≜\frac{{\hat{W}}_{MCP}\left(\omega \right)}{{\hat{W}}_{MCP}\left(0\right)},$$

where Ŵ_*MCP*_(ω) is the Fourier magnitude spectrum of ω_*MCP*_(*t*) and [0, ω_*c*_] is the frequency band where active movement is considered to occur.

In this study, ω_*c*_ was set to be 4π rad/s, or 2 Hz, which covered active movements of the participants in this study. The angular velocity of the MCP joints was obtained through numerical differentiation of the angle of the MCP joints and zero-phase filtering with a 4th-order, 2-Hz Butterworth low-pass filter.

A one-way repeated-measures analysis of variance (ANOVA), with trial (three trials) and motion (flexion and extension) as the independent variables, was used to evaluate performance changes across repeated measurements. If the sphericity assumption in ANOVAs was violated, then Greenhouse-Geisser adjusted *p*-values were used. All analyses were preceded by Shapiro–Wilk tests of normality and their results were employed unless the data were highly skewed. A one-way ANOVA is considered as a robust test against the normality assumption.

For delays in initiation and termination of the antagonist muscle, we excluded the cases that the response of the antagonist muscle looked like noise (i.e. the activation level did not remain above the threshold for more than 0.1 s) and that activation of the antagonist muscle was made after de-activation of the agonist muscle from analysis.

Nonparametric correlation Spearman's rank analyses of movement smoothness with delays in initiation and termination, antagonist activation ratio, and torque generation were performed to investigate whether those factors considered in this study influenced movement smoothness.

The statistical analyses were performed with SPSS version 20.0 (SPSS Inc., Chicago, USA). The significance level was set at 0.05.

## Results

**Figure 2:**
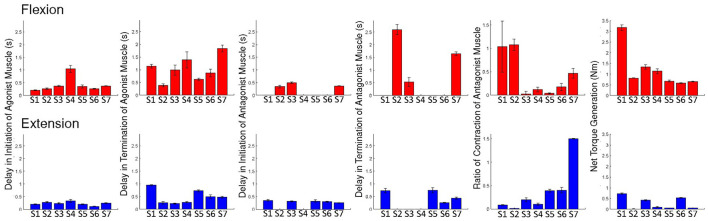
Individual results: the delays of initiation and termination of the agonist muscle, delays of initiation and termination of the antagonist muscle, and ratios of antagonist muscle activation and the net torque, during voluntary flexion and extension. Error bars are ± 1 standard error of the mean.

First of all, in the case of delays in initiation and termination of the antagonist muscle, we excluded four participants from analysis for the flexion trials, while we excluded two participants for initiation delay and three participants for termination delay from analysis for the extension trials (refer to Figure 2). De-activation of the antagonist muscle in one subject (S3) ended before the cues of termination. Considering our small sample size, delays in initiation and termination of the antagonist muscle were excluded from ANOVA. Repeated-measures ANOVAs were applied to delays in initiation and termination of the agonist muscles, antagonist activation ratio, and torque generation.

ANOVA on delay of initiation of the agonist muscle showed a marginally significant main effect of motion [*F*_(1, 6)_ = 5.016; *p* = 0.066; $\eta_{p}^{2}$ = 0.455]. We found no significant main effect of trial (*p* > 0.1) and no significant interaction effect (*p* > 0.1). These results imply that activation of EDS is marginally faster than FDS in response to auditory cues and that there is no learning effect as trials advanced. ANOVA on delay of termination of the agonist muscle showed a significant main effect of motion [*F*_(1, 6)_ = 7.070; *p* = 0.038; $\eta_{p}^{2}$ = 0.541]. No significant main effect of trial (*p* > 0.1) and significant interaction effect (*p* > 0.1) were reported. These results imply that de-activation of EDS is faster than FDS and that there is no learning effect. ANOVA on the ratio of antagonist muscle activation reported no significant main effects and interaction (*p* > 0.1).

ANOVA on net torque generation showed a significant main effect of motion [*F*_(1, 6)_ = 10.770; *p* = 0.017; $\eta_{p}^{2}$ = 0.642]. No significant main effect of trial (*p* > 0.1) and significant interaction effect (*p* > 0.1) were reported. These results imply that greater net torque was produced during flexion than during extension and that there is no learning effect.

**Figure 3:**
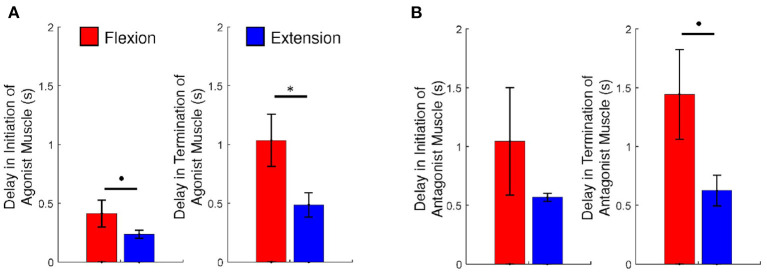
**(A)** The delays of initiation and termination of the agonist muscle and **(B)** the ratios of antagonist muscle activation and the net torque, during voluntary flexion and extension. An asterisk indicates statistical significance (*p* < 0.05) and a dot indicates marginally statistical significance (*p* < 0.1). Error bars are ± 1 standard error of the mean.

Figure 3 depicts a summary of the ANOVA results.

**Figure 4:**
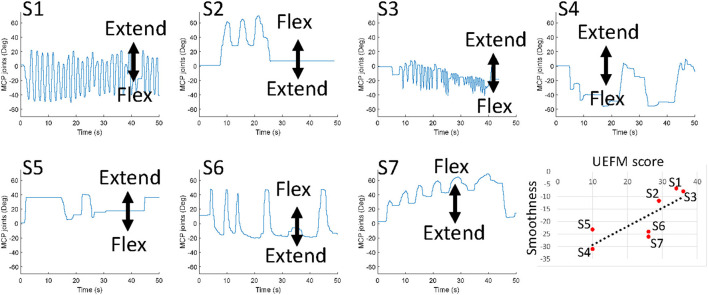
Free movements of the MCP joints of individual participants. The last plot presents the trend of movement smoothness versus the total UEFM score.

Figure 4 exhibits movements of the MCP joints of individual participants and the trend of movement smoothness versus the total UEFM score. Correlation analysis showed that movement smoothness was correlated with the total UMFM score (*p* = 0.031, *r* = 0.800). We found that movement smoothness was not significantly correlated with any other factors we investigated in this study (*p* > 0.1). The results would mean that delays of initiation and termination of flexors and extensors, ratio of antagonist muscle activation, and the net torque generation do not affect movement smoothness.

## Discussion

Originally, corticospinal motor inputs in people without neurological damage are nearly equally distributed between flexors and extensors (32). But stroke takes the ability to maintain their posture at neutral away from the victims. While victims with the parietal lobe lesioned are likely to hold their fingers extended and have difficulty in flexing their fingers (33), victims with a damaged corticospinal system are likely to have their fingers flexed and show difficulty in extending their fingers (34). In this study, we investigated activation/de-activation timing of the flexor and extensor of the MCP joints on the paretic side in stroke survivors with severe-to-moderate impairment as well as force generation in an effort to identify reasons of asymmetric motions. It would be reasonably speculated that severe-to-moderate impairment is related to the use of alternative motor pathways (i.e., RST), following CST damage. Though reasons of asymmetry in extensors and flexors could be found in spasticity (4) and altered motor coordination (35, 36), we focused on changes in neurological mechanisms of flexors and extensors following stroke presumably under the use of alternative motor pathways. We conducted isometric evaluation for the timing test to minimize the influences of spasticity and altered motor coordination.

Above all, we observed that participants generated a greater amplitude of torque at the MCP joints during flexion than during extension (*p* < 0.05), as seen in Figures 2, 3. These results would mean that all participants might have a deficit in generating corticospinal system inputs (34, 37). It is hypothesized that those participants may employ the RST in an adaptive manner. The use of the RST results in increased flexor activity, by increasing the resting potential of the motoneurons up to the threshold (21). The RST is even hypothesized to suppress extensor activity (19, 20). This might be why the net torque is generated greater during flexion in comparison to during extension.

We would have hypothesized that the degree of activation of the antagonist muscle or FDS increases during extension, while that of the antagonist muscle or EDS decreases durng flexion due to the influence of the use of the RST. But this is not the case for our results. We observed that the activation level of FDS during extension versus that exhibited during flexion is not significantly different than the activation level of EDS during flexion versus that exhibited during extension (*p* > 0.1), as presented in Figure 3. A previous study suggested that persistent and inappropriate flexor activation plays a role in limiting voluntary MCP joint extension (4). However the study did not present a comparison with extensor activation during voluntary MCP joint flexsion; it would not be reasonable to assert that difficulty in extension merely originated from eccessive activation of the flexor. This study revealed that the extensor of the MCP joints is also activation during flexion. However, we should note that the strength of force generated by the flexors of the MCP joints is different from those by the extensors of the MCP joints. Asymmetry in torque generation could be attributed to the weakness of the extensor muscles (4, 23). The magnitude of torque that the MCP joint extensors generate is typocally too small to overcome that generated by the MCP joint flexors during extension.

We found that movement smoothness was correlated with the total UMFM score (*p* = 0.029). Movement smoothness is an indicator of motor coordination in individuals with stroke that covers the activation timings of the muscles in the antagonistic setup as well as the activation strength of the muscles. Typically movement smoothness improves with recovery following (30, 38). Generally individuals with greater UEFM scores look smoother sinusoidal motions at the MCP joints in comparison to individuals with lower UEFM scores. These results might be linked to CST integrity, since improved movement smoothness requires sufficient controllability or/and strengths of individual muscles (39–41).

However, we found that movement smoothness was not influenced by activation/de-activation delays and activation ratio of the antagonist muscle which we have focused on (refer to Table 2). It was demonstrated that muscle weakness is the main contributor to difficulty in extending joints (40). Extensor weakness leads to the lack of torque enough to overcome torque produced by flexors in addition to strong spasticity and increased stiffness of flexors of the MCP joints (4, 40). Our finding enhances evidence that the weakness of extensors for the MCP joints is the main reason of asymmetry between flexors and extensors.

**Table 2:** Results (Spearman's rank correlation coefficient and *p*-value) of correlation analysis of the smoothness of MCP joint movement with the total UEFM scores, delays in initiation and termination, antagonist muscle ratio, and net torque, during voluntary flexion and extension, respectively.

	**UEFM**	**Initiation of FDS (flexion)**	**Termination of FDS (flexion)**	**Antagonist ratio (flexion)**	**Net torque (flexion)**	**Initiation of EDS (extension)**	**Termination of EDS (extension)**	**Antagonist ratio (extension)**	**Net torque (extension)**
Smoothness	*r* = 0.804, *p* = 0.031	*r* = −0.536, *p* = 0.215	*r* = −0.393, *p* = 0.383	*r* = 0.143, *p* = 0.760	*r* = 0.607, *p* = 0.148	*r* = −0.378, *p* = 0.403	*r* = 0.107, *p* = 0.819	*r* = −0.500, *p* = 0.253	*r* = −0.286, *p* = 0.535

The highlighted finding of this study is perhaps that the termination of EDS during extension is significanly faster than that of FDS during flexion (*p* < 0.05). Also we found that five of the seven participants showed faster activation of EDS than FDS, in response of auditory cues (see Figure 2). Even the remaining participants (S1 and S2) did show a small difference between intiniation delays of the flexor and extensor. We would have hypothesized that the activation and de-activation of the flexor are faster at least in comparison to the extensor, since the prevailing tract, the RST, favors and facilitates flexors. Significant differences between intiniation and termination delays of the flexor and extensor would indicate the existence of separate cortical sources and neural pathways for the extensors of the MCP joints. As mentioned above, our results are relatively opposite to the theory that the use of the RST results in eccessive activation of the flexor during voluntary extension. The theory natually leads to an assumption that the cortical source and neural pathway for the extensors of the MCP joints are strongly coupled with those for the flexors of the MCP joints, in particular, timewise. But significant differences in intiniation and termination delays nullify the assumption.

Differences in intiniation and termination delays might be due presumably to different neural routes to the motor neuron pools from the brain. The first plausible explaination about different neural routes can be found in the hypothesis that MCP flexors are predominantly governed by the contralesional RST, while MCP extensors are predominantly governed by the ipsilesional RST, in stroke patients with severe-to-moderate impairment (12, 37, 42, 43). In the case of extension, the extension synergy expression could be influenced by the vestibulospinal tract (44) and an output from the contralesional RST that facilitates ipsilateral extensors rather than flexors (43, 45); MCP extension could be conducted through different channels. If this hypothesis works, there is surely a clear difference in pathways for extensors and flexors of the MCP joints. To activate MCP flexors, motor command goes down to the motor neuron pool through the contralesional RST. Meanwhile, to activate MCP extensors, motor command goes down to the motor neuron pool through the ipsilesional RST.

The second plausible explaination about different neural routes that results in differences in intiniation and termination delays could be that MCP flexors and extensors have different pathways between the motor neuron pools and the RST. It is known that the median nerve innervates MCP flexors while the radial nerve innervates MCP extensors (4, 46). A study suggested that the preferential uses of the RST and CST vary depending the severity of impairment following a stroke (27). Even if motor drives to flexors and extensors of the MCP joints are conveyed through the (ipsilesional) CST, different neural routes could explain differences in intiniation and termination delays.

Limitations of this study can be addressed. The primary limitation of this study is the small sample size. Our experiment was suspended due to the COVID-19 pandemic. We were unable to continue to collect data from stroke surviviors, as well as, individuals with no neurological disorders for comparison. Although our sample size would be thought to not be enough to make a firm conclusion that could be generalized over the whole stroke population, we conducted within-subject analyses to minimize the effect of the small size. Flexion trials always preceeded extension trials. We would have assumed that a learning effect might be influncial on experimental results. However, repeated-measures ANOVA found no main effects of trial across the measures evaluated in this study. This means that the learning effect did not affect the results of the experiment in the study. Through this study, we presented the possibility that extensors respond faster than flexors for the metacarpophalangeal joints following stroke.

This study relies on inference on CST integrity based on UEFM scores, without support of a neurophysiological study. However this inference is grounded in the general consensus about uses of the CST and its alternative tracts versus UEFM scores. We assume that it would be clear that dominance of tracts varies with the UEFM score; the CST is dominant in individuals with the range of high UEFM scores while the CRST is dominant in individuals with the range of low UEFM scores. We will be open to neurophysiology studies using transcranial magnetic stimulation (TMS) and diffusion tensor imaging (DTI) to probe CST integrity in follow-up studies.

Severe-to-moderate stroke often involves flexor spasticity or stretch reflex-related flexor activation (4, 47, 48). Originally spasticity is defined as a velocity dependent hyperactive stretch reflex (49, 50). In a study that strived to differentiate the flexion synergy from flexor spasticity, it was revealed that flexor spasticity appears only relevant during unnaturally occurring passively supported movement (51). Rather the flexion synergy is the predominant contributor to reaching dysfunction. In accordance with those findings in the previous studies, our main findings from differences in intiniation and termination delays of flexors and extensors can be regarded free from spascity. We investigated delays in the isometric condition. We cannot rule out the influence of spasticity on our finding about movement smoothness. However, our study added evidence that extensor weakness is one of the contributor to movement smooth degradation in individuals with severe-to-moderate impairment following stroke.

## Data availability statement

The raw data supporting the conclusions of this article will be made available by the authors, without undue reservation.

## Ethics statement

The studies involving human participants were reviewed and approved by Institutional Review Board of the University of Maryland, Baltimore. The patients/participants provided their written informed consent to participate in this study.

## Author contributions

DK: conceptualization, data collection, analysis, and manuscript drafting. RB: conceptualization and data collection. KK: data collection. L-QZ: conceptualization and administration. All authors contributed to the article and approved the submitted version.
